# The standards of reporting trials in pets (PetSORT): Explanation and elaboration

**DOI:** 10.3389/fvets.2023.1137781

**Published:** 2023-03-30

**Authors:** Jan M. Sargeant, Audrey Ruple, Laura E. Selmic, Annette M. O'Connor

**Affiliations:** ^1^Department of Population Medicine, Ontario Veterinary College, University of Guelph, Guelph, ON, Canada; ^2^Department of Population Health Sciences, Virginia-Maryland College of Veterinary Medicine, Virginia Tech, Blacksburg, VA, United States; ^3^Department of Veterinary Clinical Sciences, College of Veterinary Medicine, The Ohio State University, Columbus, OH, United States; ^4^Department of Large Animal Clinical Sciences, College of Veterinary Medicine, Michigan State University, East Lansing, MI, United States

**Keywords:** animal reporting guidelines, animal health, randomized trials, small animal clinical trials, companion animals

## Abstract

Well-designed randomized controlled trials (RCTs) provide the best evidence of the primary research designs for evaluating the effectiveness of interventions. However, if RCTs are incompletely reported, the methodological rigor with which they were conducted cannot be reliably evaluated and it may not be possible to replicate the intervention. Missing information also may limit the reader's ability to evaluate the external validity of a trial. Reporting guidelines are available for clinical trials in human healthcare (CONSORT), livestock populations (REFLECT), and preclinical experimental research involving animals (ARRIVE 2.0). The PetSORT guidelines complement these existing guidelines, providing recommendations for reporting controlled trials in pet dogs and cats. The rationale and scientific background are explained for each of the 25 items in the PetSORT reporting recommendations checklist, with examples from well-reported trials.

## Introduction

Of all the primary study designs, well-designed randomized controlled trials (RCTs) provide the highest level of evidence for evaluating the efficacy of interventions, when it is ethical and feasible to assign study units to intervention groups. However, RCTs with suboptimal methods may have biased results, often exaggerating the apparent benefits of the intervention (1–3). Biased trials have the potential to negatively impact decision-making by clinicians, researchers and policy makers.

Evaluating the methodological rigor of a RCT requires that the methods and results be comprehensively reported. Additionally, readers of a RCT need to be provided with sufficient information to reproduce the methodology (including the intervention) and evaluate the external validity of the conclusions, should they wish to apply that intervention in their own setting. Researchers using the results of clinical trials for systematic reviews and meta-analyses or to develop clinical practice guidelines also need the RCT to be comprehensively and consistently reported. However, many RCTs do not report all of the essential information. To address this concern for trials conducted in humans, the Consolidated Standards of Reporting Trials (CONSORT) was published in 1996, and updated in 2001 to provide recommendations for reporting of parallel-group RCTs in human healthcare. The CONSORT Statement, created by an expert consensus process, comprises a 25-item checklist and a figure to illustrate the flow of participants through a RCT (4). An accompanying explanation and elaboration document provides examples from the published literature and provides the rationale and scientific background for each of the items (5). There is empirical evidence that CONSORT has resulted in improved reporting of RCTs in human healthcare (6). Although CONSORT is intended for use in parallel-group designs, there are a number of extensions to the CONSORT statement for other designs, including extensions for cluster-randomized trials (7), crossover trials (8) and for non-inferiority and equivalence trials (9). A complete list of CONSORT extensions, and links to publications, is available at http://www.consort-statement.org/extensions.

Trials conducted in animals include some unique aspects requiring certain nuanced differences in reporting compared with trials in humans. The ARRIVE Guidelines 2.0 for *in vivo* animal experiments distinguish between essential and recommended items for reporting (10). Although ARRIVE 2.0 included examples from trials involving dogs, the examples related to pre-clinical trials in animal models of human diseases. In livestock populations, major differences include the housing of animals in groups (such as pens and barns), such that observations on individual animals are not statistically independent, and the common use of deliberate disease induction models in the species for which the intervention is intended (11). Additional differences include the use of different outcome domains compared with human trials (e.g., production and welfare domains) and nuanced differences such as the use of animals and caregivers (where “caregivers” include owners of pets or custodians of shelter animals) rather than participants. To address these differences, and to provide livestock-specific context, the REFLECT Statement was published in 2010 and comprised a method and process publication (11) and an explanation and elaboration publication (12). There is some empirical evidence that reporting has improved since 2010 in swine trials (13) and in beef trials (14), although there still is a need for improvement (13–15).

Despite the existence of reporting guidelines for RCTs, the reporting of RCTs in pet dogs and cats remains suboptimal. An evaluation of 100 clinical trials conducted in dogs and cats published between 2006 and 2008 noted substantial deficiencies in reporting (16), based on comparison to reporting recommendations in the 1996 CONSORT Statement (17). Intervention effects were more likely to show a significant benefit in trials where the method used to generate the random allocation sequence, the use of blinding, the inclusion criteria for study subjects, baseline differences between intervention groups, the measurement used for all outcomes, or possible study limitations were not reported (16). An updated evaluation of reporting quality was conducted on 196 clinical trials published in 2019 in dogs and cats (18). This evaluation, which included both parallel and crossover trial designs, still found extensive deficiencies in reporting. Therefore, a reporting guideline specific to clinical trials conducted in pet dogs and cats was developed by expert consensus. The PetSORT guidelines comprise a 25-item checklist (Table 1) and, unlike CONSORT and REFLECT, explicitly include reporting of both parallel and crossover trial designs. A description of the process and methods for developing the PetSORT guidelines is available elsewhere (19). The objective of this explanation and elaboration document is to provide the rationale and background for each item in the PetSORT reporting guidelines, and to provide examples of how each item might be reported. The examples are from published trials conducted in pet dogs and cats.

**Table 1:** PetSORT checklist of information to include when reporting a randomized trial.

**Section/topic**	**Item no**	**Checklist item**	**Reported on page no**
**Title and abstract**			
	1a	Identify the study as a randomized trial in the title.	
	1b	Summarize the objective, trial design, primary outcome(s), study population, intervention, results, and conclusions/clinical relevance.	
**Introduction**			
Background and objectives	2a	Give scientific background and explanation of rationale.	
	2b	Specify objectives or hypotheses.	
**Methods**			
Trial design	3a	Describe trial design (such as parallel, factorial, crossover) and the level of allocation of the intervention (such as animal, litter, kennel). For crossover trials, description of the number and duration of intervention and washout periods.	
	3b	Report any changes to methods after trial commencement (such as eligibility criteria), with reasons.	
Participants	4a	Report eligibility criteria for animals and their caregivers (includes owners of pets and custodians of shelter animals) at all organizational levels (such as animal or veterinary clinic). State whether animals were shelter-owned or client-owned.	
	4b	Describe settings and locations where the data were collected. Describe sources of clustering (such as multiple veterinary practices or group housing).	
Interventions	5	Describe interventions for each group with sufficient details to allow replication. Describe the unit of allocation (such as body part (eye), individual animal, litter).	
Outcomes	6a	Completely define pre-specified primary and secondary outcome measures, including how, when, and by whom they were assessed.	
	6b	Describe any changes to trial outcomes after the trial commenced, with reasons.	
	6c	If the outcome of interest (such as survival time) could be differentially impacted by euthanasia, describe methods used to reduce bias in study results (such as standardized criteria or counseling for euthanasia).	
Sample size	7a	Provide a sample size calculation or a justification for the sample size if a calculation was not performed.	
	7b	When applicable, explain any interim analyses and stopping guidelines.	
**Randomization:**			
Sequence generation	8a	Describe the method used to generate the random allocation sequence.	
	8b	Describe the type of randomization and include details of any restriction (such as stratification, blocking, and block size) used.	
Allocation concealment	9	Describe the steps taken to conceal the allocation sequence until interventions were assigned.	
Implementation	10	Describe who generated the random allocation sequence, who enrolled study subjects, and who assigned them to interventions.	
Blinding or masking	11a	Report which individuals (such as caregivers, investigators, outcome assessors, data analysts) were blinded/masked after allocation. Provide justification if not blinded/masked.	
	11b	If relevant, describe the similarity of interventions.	
Statistical methods	12a	Describe the statistical methods used to compare groups for primary and secondary outcomes.	
	12b	Describe the methods used for ancillary analyses, such as subgroup analyses and adjusted analyses; report if these were pre-specified in the protocol or unplanned.	
**Results**			
Study subject flow	13a	For each group, state the number of study units (body part, individual animal, or litter) that were assessed for eligibility, randomly assigned, received the intended intervention, and were analyzed for each primary and secondary outcome.	
	13b	Quantify and explain any losses and exclusions after randomization for each group (such as the number per group removed due to adverse events) and for each intervention period in a crossover trial.	
Recruitment	14a	Report the dates defining the periods of recruitment and follow-up.	
	14b	If the trial was discontinued early, provide the reason.	
Baseline data	15	Provide a detailed description (such as a table) of baseline demographic and clinical characteristics that could impact the outcomes for each group.	
Numbers analyzed	16	Report the number analyzed for the primary and all secondary outcomes and whether the analysis was by original assigned groups (intention-to-treat) or per-protocol. Explicitly report the numbers of units lost to follow-up and, if relevant, the number of animals with changed intervention assignments (if relevant for per-protocol).	
Outcomes and estimation	17a	For each primary and secondary outcome, report the results for each group, and the estimated effect size and its precision (such as 95% confidence interval).	
	17b	For binary outcomes, present both absolute and relative effect sizes.	
Ancillary analyses	18	Present the results of any other analyses performed, including subgroup analyses and adjusted analyses, distinguishing pre-specified from unplanned or exploratory analyses.	
Harms	19	Describe the methods for detection of adverse events and report all adverse events (expected, unexpected, and suspected) or unintended effects observed in each group or their absence.	
**Discussion**			
Interpretation	20	Ensure that interpretation is consistent with results, balancing benefits and harms, and considering other relevant evidence.	
Generalizability	21	Discuss generalizability (external validity, applicability) of the trial findings.	
Limitations	22	Discuss trial limitations, addressing sources of potential bias, imprecision, and, if relevant, multiplicity of analyses. Consider potential carryover effects if a crossover trial.	
**Other information**			
Registration	23	State whether the trial was registered and, if so, provide a registration number and name of trial registry. If not, provide a reason for not registering the trial in advance.	
Protocol	24	State if the full trial protocol was finalized a priori and where it can be accessed. Describe any protocol deviations with justification.	
Funding and transparency	25	State sources of funding and other support (such as supply of drugs), role of funders, conflict of interest, ethical approval for human (if applicable) and animal subject use, and quality standards used.	

## PetSORT checklist items

For the examples included in this manuscript, square brackets ([]) indicate where explanatory information has been inserted into the quoted text by the PetSORT authors to clarify the text used in the example. When sections of the quoted text have been removed for brevity, the PetSORT authors have included “….” to indicate that the original text was truncated. Citations included in the quoted text have been removed for clarity.

### Title and abstract

***Item 1a. Identify the study as a randomized trial in the title.***

**Example:**

“*Effect of targeted pulsed electromagnetic field therapy on canine postoperative hemilaminectomy: A double-blind, randomized, placebo-controlled clinical trial.”* (20)

**Explanation:**

Including the method of allocation of animals to groups in the title allows a reader to rapidly determine the study design, which might facilitate a decision whether to read the article. Individuals conducting systematic reviews might restrict reviews to randomized controlled trials (RCTs), so including this information in the title facilitates rapid screening or screening using artificial intelligence. Finally, including words like “random allocation,” “randomized” or “randomization” in the title aids indexers in correctly identifying the study design for the meta-data in electronic databases (21). In a review of 196 trials in dogs or cats, 36 included a description of randomization in the title, although 178 of the trials reported random allocation in the methods section (18).

***Item 1b. Summarize the objective, trial design, primary outcome(s), study population, intervention, results, and conclusions/clinical relevance.***

**Example:**

“*Background: Rabacfosadine (RAB, Tanovea-CA1) is a novel chemotherapy agent conditionally approved for the treatment of lymphoma in dogs*.

*Hypothesis/Objectives: To determine the efficacy and safety of RAB in dogs with lymphoma*.

*Animals: One hundred and fifty-eight client-owned dogs with naïve or relapsed multicentric lymphoma were prospectively enrolled from January to October 2019*.

*Methods: Dogs were randomized to receive RAB or placebo at a 3:1 ratio. Treatment was given every 21 days for up to 5 treatments. Study endpoints included progression-free survival (PFS), overall response rate (ORR) at a given visit, best overall response rate (BORR), and percent progression free 1 month after treatment completion. Safety data were also collected*.

*Results: The median PFS was significantly longer in the RAB group compared to placebo (82 vs 21 days; P* < *0.0001, HR 6.265 [95% CI 3.947-9.945]). The BORR for RAB-treated dogs was 73.2% (50.9% complete response [CR], 22.3% partial response [PR]) and 5.6% (0% CR, 5.6% PR) for placebo-treated dogs (P* < *0.0001). One month after the last treatment, 37 RAB-treated dogs (33%) were progression free compared with no placebo-treated dogs (P* < *0.0001). The most common adverse events observed in the RAB group were diarrhea (87.5%), decreased appetite (68.3%), and vomiting (68.3%) and were generally low grade and reversible. Serious adverse events were reported in 24 RAB-treated (20%) and 5 placebo-treated dogs (13%)*.

*Conclusions and Clinical Importance: Rabacfosadine demonstrated statistically significant antitumor efficacy in dogs with lymphoma when administered every 21 days for up to 5 treatments as compared to placebo*.” (22)

**Explanation:**

The abstract of a journal article should provide sufficient information to allow the reader to decide whether to read the full article. The information should accurately reflect the methods and results of the study, since some individuals might not have access to the full text of the article. The abstract should not include information that is not reported in the article nor should it ignore any important harms identified during the study. Although there is evidence from studies of human trials that structured abstracts are generally better in quality and more informative than narrative abstracts (23, 24), the decision on structure and length of an abstract is typically decided by the journal style. An extension of the CONSORT statement for reporting of abstracts is available, which provides recommendations for comprehensive reporting of RCT abstracts (25, 26). Illustrative examples of how the recommended guidelines can be adhered to with the short word limits allowed for some abstracts are available for human healthcare examples (27). When evaluated against these guidelines, there is evidence that reporting of abstracts of RCTs is suboptimal in veterinary medicine (28) and specifically in trials conducted in dogs and cats (18).

### Introduction

#### Background and objectives

***Item 2a. Give scientific background and explanation of rationale.***

**Example:**

“*Lymphomas are among the most common cancers in dogs, having an annual incidence rate of approximately 24 cases/100,000 dogs at risk [reference]……… Among treatment-related factors that may affect the prognosis for dogs with nodal lymphomas, treatment with prednisone or other glucocorticoids prior to initiating cytotoxic chemotherapy repeatedly has been reported to have a deleterious effect on remission rate and survival time [reference]. The pathogenesis of this clinical phenomenon is not fully understood; however, it has been hypothesized that corticosteroid treatment upregulates expression of the cell membrane–associated drug efflux pump P-glycoprotein, conferring multidrug resistance [reference]…… Although prior treatment with prednisone is reportedly strongly correlated with poor outcome in dogs with chemotherapy-treated lymphomas [reference], the effect of omitting prednisone from the CHOP chemotherapy protocol on treatment outcome has undergone limited investigation……. Zandvliet et al. [reference] recently reported that omission of prednisolone from a CHOP-based chemotherapy protocol did not have a significant effect on remission rate or progression-free survival time in dogs with nodal lymphomas. These authors, however, did not enroll their target sample size; as such, the trial may have been underpowered to detect significant differences in clinically important outcome variables between study groups. Furthermore, to our knowledge, that study represents the only report to date describing the effect of prednisone omission from CHOP chemotherapy, and the results have not been reproduced by others*.

*Therefore, the primary objective of the study reported here was to determine the effect of prednisone omission from a CHOP-based chemotherapy protocol on the median progression-free survival time of dogs with histopathologically confirmed peripheral nodal lymphomas.”* (29)

**Explanation:**

The introduction provides the contextual background to the trial and should include a description of the problem that will be addressed, as well as the theoretical basis of action for the proposed intervention. The introduction section also should provide a justification for the need for conducting the trial and an indication as to how the trial results will enhance knowledge. This information should be grounded in the available literature, either with reference to other published trials or to a systematic review on the topic, if available.

***Item 2b. Specify objectives or hypotheses.***

**Examples:**

“*The purpose of this study was to investigate the use of a 100% pure medical-grade honey (MedihoneyTM) as an alternative to topical antimicrobials in the control of canine nasal intertrigo. ……. we hypothesize that medical-grade Manuka honey would be safe and clinically superior to a placebo topical therapy at treating nasal intertrigo in brachycephalic dogs. The main objective of this study was (1) to compare the severity of intertrigo clinical signs and cytological findings before and after a 21-days treatment course with either MedihoneyTM or a honey-scented placebo hydrogel. Our secondary objectives were (2) to assess how each treatment affected the culturable microbial flora of nasal intertrigo, which is currently undefined, and (3) to record any adverse effect with either treatment.”* (30)

“*The purpose of this study was to compare a [bupivacaine liposome injectable suspension] with a control protocol in cats after ovariohysterectomy (OHE). The hypothesis was that a BLIS block would provide equivalent pain relief …. This study was designed as a randomized, double-blind, non-inferiority trial.”* (31)

**Explanation:**

The objective(s) of a trial describes the question(s) that the trial will address, including the trial purpose (superiority, equivalence, or non-inferiority), the intervention and comparison groups, the population, and the primary outcomes. The framing of the trial question, the null hypothesis being tested, will differ based on whether the purpose of the trial is to evaluate superiority of one intervention over another, equivalence of interventions, or non-inferiority of interventions (32). In an evaluation of completeness of reporting in 196 trials in dogs and cats, the objectives statement generally included a description of the intervention groups, population, and outcomes. However, the trial purpose was rarely reported; of the 196 trials, none were described as evaluating superiority or equivalence and 6 were described as non-inferiority trials (18). It is important that researchers describe the purpose of their trial, as it will impact the methods, analysis, and interpretation.

### Methods

#### Trial design

***Item 3a. Describe trial design (such as parallel, factorial, crossover) and the level of allocation of the intervention (such as animal, litter, kennel). For crossover trials, description of the number and duration of intervention and washout periods.***

**Examples:**

“*The study was a prospective parallel unmasked block-randomized controlled trial comparing two weight loss intervention groups: (1) traditional group with dietary restriction alone (n* = *9); (2) technology group that used dietary restriction, digital scales, smart feeders, activity monitors and pet treat cameras (n* = *6).”* (33)

“*A randomized crossover study was performed. Dogs received either sildenafil (1 mg/kg, PO, q 12 h) or a placebo for 14 days, followed by a 7-day washout period, then the opposite treatment for 14 days.”* (34, 35)

**Explanation:**

A number of trial designs may be used, including parallel, crossover, and cluster trials. In dogs and cats, ~75% of trials use a parallel design with the remainder using a crossover design (18). Ideally, this information should be included in the abstract (34). Additional information on reporting the less common non-inferiority and equivalence designs is available as an extension of the CONSORT statement for reporting RCTs in human populations (9).

***Item 3b. Report any changes to methods after trial commencement (such as eligibility criteria), with reasons.***

**Example:**

“*For three dogs, the investigator deemed the level of analgesia achieved by repeat boluses of 1* μ*g/kg bolus of fentanyl to be inadequate on ethical grounds and for the remainder of the monitoring period for these cases the fentanyl bolus dose was increased to 2* μ*g/kg.”* (36)

“*A number of required changes were made to study protocol at various stages, mainly because the rate of recruitment of cases was slower than expected. Firstly, the original plan was for all cases to be seen at the SATH; however, initial recruitment was slow and the major hurdle was found to be reluctance to travel. For this reason, compensation for client travel was introduced and administration by the first-opinion veterinarian was then allowed. Second, as based upon the power calculation, the initial intention was to recruit a total of 40 dogs (20 treatment and 20 controls). However, the slow recruitment meant that there were concerns that the treatments would exceed their expiry date, initial set for two years after product manufacture. As a result, two treatments were sacrificed (1 treatment, 1 control) and sent back to the manufacturer so that enzyme activity and microbial contamination could be retested, and enabling an extension to the expiry date to be granted.”* (37)

**Explanation:**

The proposed methods for a trial should be specified *a priori* in a protocol. However, there are circumstances where it is necessary to modify the research plan during the trial. Changes might be needed for ethical reasons (e.g., if there are unexpected adverse effects from an intervention), due to information obtained from new publications, or result from issues with recruitment where a change in eligibility criteria might be needed. It is important to be transparent when reporting any such modifications to the pre-specified plan, and to justify the changes so as to provide readers with appropriate context when interpreting results and conclusions. Generally, important changes to methods are poorly reported in trials of dogs and cats; in a recent study evaluating reporting of 196 trials, no information was provided on whether there were changes in 89%, and none of the studies included an explicit statement that there were no changes to the study protocol (18). For transparency, it is recommended that authors state that there were no changes, when appropriate. External quality assurance programs that monitor study procedures, including protocol deviations and amendments, are essential in regulatory, pivotal clinical trials. This high level of data integrity should be considered for non-regulatory clinical trials as well.

#### Participants

***Item 4a. Report eligibility criteria for animals and their caregivers (includes owners of pets and custodians of shelter animals) at all organizational levels (such as animal or veterinary clinic). State whether animals were shelter-owned or client-owned.***

**Examples:**

“*Client-owned dogs were recruited at a single tertiary referral veterinary hospital between April 2014 and November 2015. Dogs were considered eligible for inclusion in this trial if they were anemic, with a packed cell volume (PCV) of less than 35 percent, and if they had at least one of the following features suggestive of immune-mediated haemolysis: prominent spherocytosis on examination of a fresh blood smear by a board-certified clinical pathologist or participant in a specialist training programme, a titre of at least 1:16 in a multivalent direct antiglobulin (Coombs') test, or persistent microscopic or macroscopic agglutination of red blood cells after dilution in saline.”* (38)

“*Dogs were eligible for the study if both the owner and primary care veterinarian consented to their involvement, they were overweight (i.e. body condition score [BCS] 6–9/9), and if they had a good temperament (i.e. easy to handle, not nervous or fearful, and not aggressive to other dogs or people).”* (39)

**Explanation:**

All eligibility criteria used to define the study population should be comprehensively described in order for readers to judge the external validity of the trial. Eligibility criteria for both the animal participants and their caregivers should be reported. This is because differences between the study population and the target population can occur through the determination of which types of study participants will be excluded from the trial (in addition to issues of who consents to participate and who attends appointments). These differences may need to be taken into consideration when determining the relevance of the trial results in guiding clinical practice. It is not necessary to include both eligibility and exclusion criteria since each criterion can be phrased to either include or exclude participants (40).

***Item 4b. Describe settings and locations where the data were collected. Describe sources of clustering (such as multiple veterinary practices or group housing).***

**Examples:**

“*Eighty-one mixed-breed adult female cats (2.8* ± *0.7 kg) from three local animal shelters were admitted to the veterinary teaching hospital (Center Hospitalier Universitaire Vétérinaire) of the Faculty of Veterinary Medicine, Université de Montréal for elective ovariohysterectomy between June and October 2018*.” (41)

“*Ours was a prospective, multicenter, randomized, open-label (nonblinded) trial. Dogs with thrombocytopenia and evidence of bleeding presented to 12 study centers (8 private specialist referral practices and 4 university teaching hospitals) were screened*.” (42)

**Explanation:**

The settings and locations where trials were conducted can impact the external validity of the trial. Description of trial settings should include the geographic location(s), the month(s), and year(s) during which the trial was conducted, and the number and type of facilities from which participants were recruited (e.g., first opinion practices, referral practices, or veterinary teaching hospitals). When relevant to study conduct or outcomes, differences in management decisions that might vary across participant groups should be described. This could include differences in standard operating procedures (SOPs) within a single facility (e.g., protocols for isolation or housing that differ based upon presence or absence of a particular disease or condition) or across different facilities in a multicenter trial. Additional information on SOPs may need to be included in Supplementary material if not possible to describe within journal word limits.

Regardless of the unit of allocation, the authors also should describe the animal housing, specifically, whether the animals were individually housed or housed in a group. This information is necessary to evaluate whether the statistical analysis is appropriately controlled for any non-independence of study animals and also allows the reader to judge the external validity of the trial conditions. If animals were housed in groups, the number of animals per group should be stated. This component is particularly applicable for shelter animals or animals housed in hospital settings during a trial.

#### Interventions

***Item 5. Describe interventions for each group with sufficient details to allow replication. Describe the unit of allocation (such as body part (eye), individual animal, litter).***

**Examples:**

“*One day following baseline examination (day 2), each dog received 0.05 mL of commercially available 5% NaCl ointment (Akorn, Inc., Lake Forest, IL, USA) applied to the superior bulbar conjunctiva of the right eye (OD; treated eye). An equal amount of artificial tear (AT) ointment (Akorn, Inc.) was applied to the left superior bulbar conjunctiva (OS; control eye).”* (43)

“*15 animals (five males and ten females, totally 17 wounds) were treated with dry needle acupuncture by a small animal veterinarian certified in veterinary acupuncture (by International Veterinary Acupuncture Society, IVAS). Treatment consisted of one acupuncture treatment right after the surgery, when all the animals were still under anesthesia, using the acupuncture points LI4, LI11, GB34, SP6, ST36, GV14 and two local points 0.5 cm distal from both ends of the wound. The size of the needles were 0.25 mm* × *30 mm for dogs weighing above 10 kg and 0.20 mm* × *15 mm for dogs weighing less than 10 kg (sterile Zhou acupuncture needles, Wui- jiang Shenli Medical & Health Material C., Ltd). Sterile Han Il acupuncture 0.17* × *7 mm disposable needles (Han IL Acupuncture Needle Manufacturing Co.) were used for the local wound points. The needles were maintained in place for five minutes, except for the GV14 point, where the needle was maintained for 15 minutes. The control group consisted 14 cases (seven males and seven females, totally 17 wounds) that did not receive any post operative acupuncture treatment.”* (44)

**Explanation:**

The description of all intervention(s), including comparison groups, should be provided in sufficient detail to enable the intervention to be replicated and implemented. Authors should state how, when, and by whom the interventions were administered. It is important to report duration, dosage, and route of administration, essential processes for applying the intervention, training of interventionists if applicable, and monitoring of the application of the intervention. These features should be reported for all intervention groups. Descriptions such as “usual care,” “standard practices” or “as per manufacturers' instructions” are not adequate for replication.

For pharmaceutical interventions, authors should include (as a minimum) the names of the compound, as well as the concentration, dosage, delivery matrix and/or proprietary name, and the mode, frequency, and duration of administration. For biologic interventions (e.g., vaccines), the minimum description should include the target organism(s), whether the vaccine is modified-live, killed or autogenous, as well as a description of the active substance, and the adjuvant. The concentration per ml (if known), dose, delivery matrix and/or proprietary name, route of administration, and the frequency of administration also should be described. For surgical interventions, the minimum description should include the training level of the surgeon(s), relevant anatomic or other landmarks, the number of surgeons performing the procedure, the experience of the surgeon in performing the intervention procedure, and the peri-operative care, including the use of ancillary pre- and post-operative interventions such as antibiotics or pain medication. For dietary interventions, the minimum description should include the nutrient profile of intervention and control diets (on an energy basis) with at least proximate analysis, plus nutrients of concern for the individual study and metabolizable energy densities (noting methodology), a list of ingredients, whether the diet is a commercially-available product or formulated specifically for the study and whether foods other than the intervention diet(s) or dietary supplements were allowed. Similar information should be reported for dietary supplement trials since the underlying diet could have an impact on the results. Descriptions of minimum reporting recommendations for describing other types of interventions are available in CONSORT extensions for non-pharmacological interventions (45, 46), acupuncture (47), and herbal interventions (48).

Additional guidelines specific to the reporting of intervention groups have been developed in human healthcare. For example, the TIDieR guidelines (49) are intended to supplement item 5 (description of interventions) in the CONSORT Statement (4), and comprise a 12-item checklist for reporting interventions. A follow-up guideline document, TIDieR-Placebo offers additional considerations for reporting placebo and sham interventions (50). These extended guidelines may be relevant for reporting of intervention groups in trials of dogs and cats.

Interventions may be allocated at different levels, such as housing unit (e.g., kennel, cattery, or household), individual animal, or body part. The first example provided for this item pertains to a trial where the unit of allocation was a body part (the eye). The unit of allocation of the intervention should be clearly stated because this corresponds to the unit of randomization. For instance, the authors might state “each kennel room was randomly allocated” or “each individual animal was randomly allocated” or “within each animal, one ear was randomly allocated to receive [the intervention] with the other ear within the animal serving as a non-treated control”. If the unit of allocation is at a group level (e.g., room or multiple-animal kennel), the number of animals per group should also be described.

#### Outcomes

***Item 6a. Completely define pre-specified primary and secondary outcome measures, including how, when, and by whom they were assessed.***

**Examples:**

“*The study's primary a priori regulatory endpoint was confirmed overall response rate (CORR) from tumor assessments according to RECIST (v1.0) [reference provided] Response outcome was categorized as complete response (CR; disappearance of all target lesions); partial response (PR; 30% decrease in the sum of the longest diameters [LD] compared with baseline); progressive disease (PD; 20% increase in the sum of the LD compared with the smallest measured sum at any visit); and stable disease (SD; any change not qualifying as CR, PR or PD). CORR (yes or no) for each study dog was defined as complete response (CR) or partial response (PR) of target and nontarget lesions and no new lesions at Visit 13, and the overall response were confirmed at Visit 14 (only responses confirmed at Visit 14 were eligible to be counted) … A secondary efficacy endpoint, biologic observed response rate (BORR), often referred to as Clinical Benefit, which combines the stable disease (SD) rate with the CR and PR rate, also was assessed at Visit 13 and confirmed at Visit 14…An investigator blinded to treatment always made efficacy assessments.”* (51)

“*The primary endpoint was treatment failure and was defined prospectively in the protocol… The secondary endpoints defined prospectively in the protocol were the quality of life of the cat assessed by the owner (rated as normal, medium, poor, or very poor) and the left atrium (LA) diameter and left ventricle (LV) wall dimension on echocardiography.”* (52)

**Explanation:** All trials include at least two interventions (where one intervention might be an un-treated control), where the difference in outcomes between interventions is inferred to be the result of the intervention (53). The primary outcome is the pre-specified outcome which is the most clinically-important outcome, and should be used as the basis for sample size calculations (34). All other outcomes are referred to as secondary. In some instances, there may be more than one primary outcome; for instance, a trial may include both a health outcome and a quality-of-life outcome as primary outcomes. In this case, sample size calculations (Item 7) should be provided for both outcomes, with the larger sample size used in the trial. However, too many primary outcomes can lead to an unfocussed research question, might complicate interpretation of results if inferences differ for the different outcomes and could lead to issues of multiplicity in analyses (54). Additional outcomes, such as biomarkers, may be of interest, but should be identified as secondary outcomes, because the trial might not be powered to detect clinically-meaningful differences in these outcomes. Outcomes may reflect potential benefits of the intervention, but harmful outcomes also may be relevant (see item 19). Further, if an outcome is measured at multiple points in time, the primary time-point of interest should be identified. Additional outcomes, such as those related to unintended or unexpected consequences of an intervention (e.g., adverse events), should be identified as the trial progressed. These should be clearly described as outcomes identified after the trial initiation. All primary and secondary outcomes should be identified *a priori* in a trial protocol (see item 23). Outcomes not described *a priori* should be clearly reported as exploratory and hypothesis-generating.

Sufficient information on the outcome definition and measurement should be included to allow another researcher to replicate the study. The information necessary for replication includes providing a clear definition of each outcome, including a case definition (if disease status is an outcome) as well as describing the method used to measure the outcome, the method of obtaining samples for outcomes requiring diagnostic testing, and the methods used for any diagnostic testing. Any modifications to existing methods or tests should be described, rather than using ambiguous statements such as “with some modifications from manufacturers' recommendations.” Some outcomes have undergone a validation process where the measurement is standardized, and its performance has been evaluated. An example of this is the Liverpool Osteoarthritis in Dogs Clinical Metrology Instrument (55). If previously validated outcomes or techniques are used, authors should provide sufficient detail to allow replication, as well as a reference to the full description of the validated outcome measure or technique. Authors should describe the timing of outcome measurement, as well as information on who evaluated each outcome and the training of outcome assessors, if appropriate.

Where available, previously-validated scales should be used. Although not yet common in veterinary medicine, core outcome sets are being developed (53), for example for feline chronic kidney disease intervention trials (56) and trials on canine atopic dermatitis (57), and it is anticipated that additional core outcome sets will be developed in veterinary medicine. Core outcome sets provide a minimum set of outcomes that should be included in all trials on that topic, to better build a body of evidence across trials. It is anticipated that the number of core outcome sets will increase over time. Where available, authors should include these outcomes as a minimum, but could also include additional outcomes if relevant to their trial. If researchers decide not to include core outcomes where they are available, a justification should be provided.

***Item 6b. Describe any changes to trial outcomes after the trial commenced, with reasons.***

**Examples:**

“*During data collection a problem was encountered regarding the incubation time for the agar plates. When incubating the first sample, it was checked as initially planned after 24 hours as suggested by [reference], however no CFUs had formed on the agar plate. The student veterinary nurse performing the data collection discussed the problem with the veterinary surgeon and it was agreed that the sample should be incubated for a further 24 hours.”* (58)

**Explanation:**

Authors should report all major changes that occur after a trial is initiated to the designation of primary and secondary outcomes, the outcomes that were measured, or the measurement of the outcomes. There is evidence in the human literature that outcomes with a significant beneficial association with the intervention are more likely to be reported in a manuscript than those that are not statistically significant (59). Preferential reporting, wherein results are only included in a publication for outcomes with beneficial associations with the intervention, will mislead readers and also can introduce bias into systematic reviews and clinical trial guidelines (60). Therefore, it is important that results for all outcomes that were measured are included in the trial publication, and that any changes to the outcomes or their measurement are clearly reported. If there were no changes in outcomes, this should be explicitly stated. Changes in outcome measures are poorly described in trials in dogs and cats; only two of 196 trials explicitly stated whether outcome measures were changed (18). Unless there is an explicit statement on whether outcomes changed, or access to the initial trial protocol, it is impossible for the reader to determine whether selective outcome reporting occurred.

***Item 6c. If the outcome of interest (such as survival time) could be differentially impacted by euthanasia, describe methods used to reduce bias in study results (such as standardized criteria or counseling for euthanasia).***

**Example:**

No example was found in the published literature. Therefore, the following is a fictitious example of reporting of outcomes potentially differentially impacted by euthanasia: “*Given that it was not possible to blind study personnel and owners to intervention group, and because it was anticipated that there would be losses to follow up due to euthanasia, a standard approach to discussions with owners regarding euthanasia decisions was developed prior to the initiation of the trial. This standardized approach was followed for discussions with owners of dogs in both intervention groups.”*

**Explanation:**

In trials in dogs and cats, it is not uncommon for severely ill animals to be euthanized during the trial if their condition is worsening or unresponsive to treatment. This is an obvious difference from human trials and, therefore, euthanasia is not addressed in human healthcare reporting guidelines. Study animals subjected to euthanasia may be classified in different ways, including as losses to follow-up, unless mortality or euthanasia is a pre-defined outcome (61). Losses to follow up are addressed in item 13b. However, it may also be relevant to report any methods used to reduce bias due to decision-making regarding euthanasia and, if possible, to speculate on the likely direction of any biases. For example, if the outcome assessor is aware of the intervention allocation, it could affect how they discuss euthanasia with the caregiver or might influence the timing of discussions regarding the euthanasia. Similarly, if the owner is aware of the intervention allocation, it may influence their decision on whether, or when, to euthanize their pet. Ideally, the trial protocol should include a plan for the investigation and reporting of euthanasia that occurs during a trial and how euthanasia will be represented in data analysis and interpretation.

#### Sample size

***Item 7a. Provide a sample size calculation or a justification for the sample size if a calculation was not performed***

**Examples:**

“*The sample size was calculated based on the previous work [reference provided] using GPower 3.1.9.2 software (Franz Faul, Universität Kiel, Germany), and based on detecting a difference between the PCSO-524 group and the combination treatment group. We used an expected difference in change in PVF over time of 4.48, with a pooled SD of 3.45, and alpha and beta values of 0.05 and 0.9. This indicated group sizes of 27 would be required.”* (62)

For a non-inferiority trial:

“*Sample size was determined using the following parameters: probability of type I error (*α*): 0.05; probability of type II error (*β*): 0.20; expected success rate of group Z: 90 per cent; expected success rate of group P: 90 per cent; margin of difference (*Δ*): 15 per cent; experimental unit: one dog; weighted number in the test article group: 3; total sample size: 133 dogs, of which group Z: 100 dogs; group P: 33 dogs.”* (63)

**Explanation:**

The sample size of a trial should allow detection of a clinically-important difference between intervention groups for the primary outcome of a trial, if a difference exists. If a small difference is clinically important, then a larger sample size will be necessary. There is evidence that many small animal trials are substantially underpowered; Di Girolamo and Meursinge Reynders (64) reported a median sample size of 26 participants in trials published in veterinary journals compared with 465 participants in trials reported in human medical journals. Tan et al. (65) reported a median sample size of 33 in canine oncology trials, with a median value for the minimum detectable hazard ratio of 0.3 for survival and 0.6 for disease progression. Finally, Wareham et al. (66) reported a median sample size of 30 across 126 veterinary RCTs. Authors of small trials may inappropriately conclude that there was no difference in the outcome between intervention groups when the sample size was too small to detect clinically meaningful differences in that outcome as significant (67). Conversely, small trials that do get published may represent only those trials in which large treatment effects were detected. There is empirical evidence that trials with low power, but statistically significant effects tend to represent overestimates of the treatment effect and these estimates have low replicability across future trials (68). In trials of dogs and cats published between 2015 and 2020, and comprising both parallel and cross-over designs, only a third included a sample size calculation, with a further 10% not including a formal sample size calculation but providing some justification for the sample size (18).

Sample size calculations require the researcher to state the difference in outcomes between groups that corresponds to a clinically-meaningful difference, the desired statistical power to detect a difference, the acceptable type I error rate, and, for continuous outcomes, the expected standard deviation of the outcome (69). For crossover trials with a continuous outcome, the standard deviation should be the standard deviation of the within-participant differences (8). Authors should provide the values for the predicted outcome at baseline and a rationale for the determination of a clinically meaningful difference, with references from the literature on baseline risk, when available. This is important because the baseline value for binary values is associated with the sample size. For instance, a 5% difference in incidence risk between intervention groups could correspond to values of 5–10% (in the two groups) or could correspond to 45 vs. 50%. The latter scenario represents a smaller relative difference and, therefore, would be associated with a larger required sample size to detect a difference. For animals housed in groups (e.g., shelter animals kennelled in rooms), a design effect may be needed to account for non-independence (67). If allowances are made, in anticipation of non-compliance or withdrawals from the trial, this should be reported. The application of sample size calculations will differ for cluster-randomized trials (70) and for superiority, non-equivalence, and non-inferiority trials (71). Typically, a superiority trial would have a smaller sample size than a non-inferiority trial, and an equivalence trial would have a larger sample size than either a superiority or non-inferiority trial (32). The input of a researcher familiar with sample size calculations may be valuable, particularly for complex trials.

***Item 7b. When applicable, explain any interim analyses and stopping guidelines.***

**Example:**

“*A preplanned interim analysis was undertaken with predefined stopping criteria for convincing evidence of efficacy and safety, performed on data obtained after 80% of the initial anticipated study period was complete. Unblinding and termination of the study only occurred if deemed necessary by the data interim evaluation committee according to prespecified criteria [referenced Figure]. The committee consisted of 3 independent (to the study) persons: 1 biostatistician and 2 experts in canine cardiology. The P-value for stopping on the basis of convincing evidence of efficacy with respect to the primary endpoint was decided by appropriate statistical software [referenced] and set at P* < *0.01477.”* (72)

**Explanation:**

If clinical trials involve the sequential recruitment of animals meeting eligibility criteria, recruitment may occur over a prolonged time period. If an intervention is highly efficacious, or if the intervention causes unexpected harm, the trial may need to be stopped for ethical reasons; trials stopped for harm will prevent further adverse effects, while trials stopped for benefit will allow earlier dissemination of information on the benefits and prevent an effective therapy from continuing to be withheld from subjects in the comparison group (placebo group). However, a trial stopped early for benefit may have low power to detect even a relatively common harm.

Decisions related to when a trial should be ended (so-called “stopping rules”) should be elucidated *a priori* (i.e., at the protocol stage). Given that the decision to stop a trial early requires one or more interim analyses of the data, this raises statistical concerns related to multiple evaluations of the data because multiplicity of testing increases the probability of a type I error (rejecting the null hypothesis when it is true) (73). For instance, if a nominal *p*-value of 0.05 is used to define the false positive rate, examining the data at five interim analyses would lead to an overall false positive rate of 23% (73). Statistical approaches are available for conducting interim analyses to determine whether to end a trial early (74). However, stopping a trial early is not without risk; the STOPIT-2 working group compared the results of truncated trials with trials on the same research question that had not been stopped early, and reported an average relative risk ratio of 0.71 (0.65, 0.77), indicating that truncated trials tended to overestimate intervention effects (75).

#### Randomization: Sequence generation

***Item 8a. Describe the method used to generate the random allocation sequence.***

**Example:**

“*Dogs were randomly allocated without restriction to an anesthetic protocol group using tables generated by one of the authors (ET) with the random function of Microsoft*^®^ *Excel (Microsoft Corp., Redmond, WA, USA)*.” (36)

**Explanation:**

Randomization (i.e., random allocation to intervention groups) is essential to internal validity, because it minimizes differences between intervention groups. Inadequate randomization can lead to exaggerated estimates of intervention effects (2, 3). Study units should be assigned to groups on the basis of chance (i.e., a random process), to limit the potential for confounding to influence the study result or for selection bias in the assignment of study units to intervention groups. The term “random” has a precise meaning, wherein each study unit has a known probability of receiving a given intervention prior to the assignment of the interventions. Thus, the actual intervention that a specific study unit is allocated to is determined by a chance process and cannot be predicted. The methods used to generate the random allocation sequence should be reported in sufficient detail to allow the reader to assess the possibility of bias in group assignments. Many methods of sequence generation are adequate (random number generator using a software package or website, drawing numbers from a hat, or a coin toss), although not all of these methods are reproducible. However, simply stating that there was “random allocation” or “randomization” of subjects, without further elaboration on the exact method means that readers cannot judge the adequacy of the approach. Therefore, authors should specify the method of sequence generation to ensure readers have confidence that the method used was actually random. Deterministic allocation methods, such as alternation based on patient order or days of the week are not random, as there may be a characteristic(s) related to the outcome that are also related to the allocation method (76). If a deterministic method of allocation is used, the study is a controlled trial rather than a RCT. Regardless, the method of allocation should be clearly reported. Additionally, authors should discuss the potential for deterministic methods of allocation to create groups that differ by important characteristics related to the outcome (item 20).

***Item 8b. Describe the type of randomization and include details of any restriction (such as stratification, blocking, and block size) used.***

**Examples:**

“*For each trial site, cats were block randomized to treatment group based on order of enrollment, using blocks of 3 with a ratio of 2:1 (telmisartan:placebo).”* (77)

“*A stratified randomization scheme was used based upon a total target of 64 enrolled cats. The four strata were established upon initial client-specific outcome measures (CSOMf) of pain (“high”* = *CSOMf score 13–20; “low”* = *CSOMf score of 7–12 [see below]) and pre-study supplementation status such that the strata were: (1) high pain/on supplements; (2) low pain/on supplements; (3) high pain/not on supplements; and (4) low pain/not on supplements. Within each of the four strata, 16 consecutive case numbers were randomized in blocks of two to maintain balanced treatments.”* (78)

**Explanation:**

Simple randomization means that all animals are assigned to an intervention group by a random (chance) process without consideration of any other characteristics of the animal. Although simple randomization is used to minimize differences between groups, it does not guarantee that all important characteristics are balanced between intervention groups. There are additional randomization approaches that ensure equal sample sizes among intervention groups, or account for other variables because they are known to be strongly related to the outcome and, if such factors were unevenly assigned to groups, it could potentially bias the outcome (76, 79). Such methods are most often used when the sample size is small. Block randomization, also called permuted block randomization, is used to ensure an equal distribution of study units to intervention groups. Study units are divided into several blocks with equal or unequal sizes, and animals are randomly allocated to intervention within blocks. For example, in a study of 32 animals, there may be eight blocks of four animals with an equal number of intervention and control study units allocated in each block. Essentially, a block creates a group with its own randomization schedule. Similarly, studies that assign interventions within animals use the animal as a block. For example, a study might be allocating intervention to eyes and, for each dog, a coin flip might determine which eye will receive the intervention, and which receives the control; in such a scenario, the dog is a block and simple randomization occurs within the block. Finally, different clinicians (or different sites in a multi-center trial) might also be blocks for the purposes of randomization, if researchers believe that between-clinician differences (or between center differences) might impact the outcome.

Stratified randomization can be used to minimize differences between groups for variables that are strongly related to the outcome. With this method, study units are randomly allocated to intervention groups within strata of the variable (80). For instance, if investigators are concerned that sex could be strongly related to the outcome, they may stratify the study population into males and females, and then randomly allocate to intervention groups within each sex stratum. Sometimes, the factor related to the outcome is a continuous variable, such as age and weight. An example would be when the success of an orthopedic surgical procedure might be impacted by weight; in such a circumstance, it would be desirable to balance weight across the interventions. One option is to create categories based on a threshold; for example, a simple randomization schedule could be used for animals above 25 kg (55 lb), a different schedule used for dogs between 15 and 25 kg (33 and 55 lbs), and a third used for dogs <15 kg.

#### Allocation concealment

***Item 9. Describe the steps taken to conceal the allocation sequence until interventions were assigned.***

**Examples:**

“*Order of treatment was assigned by use of sealed envelopes; half of the dogs received IV administration of dexmedetomidine followed by OTM administration, and the other half of the dogs received the treatments in the opposite order.”* (81)

“*The randomization schedule and key were maintained by the pharmacy personnel and not disclosed to investigators until completion of the statistical analysis.”* (82)

**Explanation:**

Allocation to intervention requires two steps: enrollment into the study based on eligibility criteria and assignment to intervention. Both of these steps should occur without the investigator or caregiver having knowledge of the intended allocation (i.e., allocation concealment). Therefore, this item covers the methods used to ensure that the individual enrolling participants is blinded during the enrollment process. A common approach is to have assessment for eligibility be conducted by a different person than the one creating the random sequence. For example, the third party could create envelopes or computer access codes that are only accessed after all steps of eligibility and enrollment are completed.

In veterinary science, it is rare to report concealment of allocation prior to enrollment (64), but such reporting should be encouraged. Concealing the allocation of interventions reduces the potential for prognostic factors to influence enrollment into the trial, thereby circumventing randomization (76). If prognostic factors influence enrollment, the source population (i.e., those eligible to be enrolled) might differ from the study population (i.e., those actually enrolled) and the effect size could be biased. Allocation concealment also is important to ensure external validity of the results: if specific animal types or those with differing disease severity are excluded from enrollment based on those characteristics, the resulting study population will not represent the source population. For example, in a trial where the entire spectrum of dogs with diabetes mellitus (controlled and uncontrolled) were eligible, if allocation was not concealed, the person assessing eligibility might “steer” animals with uncontrolled diabetes away from the trial for fear that an uncontrolled animal might receive an inferior intervention.

#### Implementation

***Item 10. Describe who generated the random allocation sequence, who enrolled study subjects, and who assigned them to interventions.***

**Examples:**

“*All owners and dogs attended an enrolment visit at the SATH during the same 2-day period in January 2017. During this visit, each dog had a 30-min individual consultation with two of the study investigators, one of whom was an European Board of Veterinary Specialists European Veterinary Specialist in Small Internal Medicine (AG), and the other (GW) was a Royal College of Veterinary Surgeons Registered Veterinary Nurse (RVN)*.” (82)

“*At each site, an unblinded treatment administrator allocated animals to a group, and administered the DOCP. This person was not involved in animal enrolment or assessment, and had sole access to the randomisation sequence.”* (63)

**Explanation:**

This item relates both to the accountability of the described random sequence allocation and allocation concealment procedures. The information enables the reader to assess the feasibility of the concealment process. For example, if the authors report that the same person generated the sequence, assessed eligibility, and assigned allocation, the risk of bias may be greater than if different people performed these tasks. The ultimate goal of detailed information covered in both Items 9 and 10 is to make transparent the allocation procedure by providing explicit detail. Further, by identifying who did what tasks, there is increased accountability for reporting the allocation methods.

#### Blinding or masking

***Item 11a. Report which individuals (such as caregivers, investigators, outcome assessors, data analysts) were blinded/masked after allocation. Provide justification if not blinded/masked.***

**Examples:**

“*…the investigators, owners and statistician were blinded to the treatment allocation.”* (83)

“*Clinicians were not blinded, which could have allowed some bias in toxicity and response assessment, but the response and toxicity endpoints were designed to be as objective as possible to minimize bias.”* (84)

**Explanation:**

Blinding of caregivers, outcome assessors, and data analysts can prevent bias in trial results. For example, if caregivers, nursing staff, or clinicians are aware of which animals received which intervention, then they may care for the animals differently and this could impact the outcome. At outcome assessment, if the outcome assessor is aware of which animals received which intervention, and the measurements are subjective, then outcome assessment might be influenced by knowledge of the intervention. During data analysis, if the intervention is known, then subjective assessment of aspects of the analysis such as transformations or removal of outliers could be influenced by knowledge of the intervention group. Authors should clarify when blinding is used and exactly how it was employed. The terms single-, double-, and triple-blinded should not be used as there is no standard for their use especially in veterinary science (85). Instead, it is important to clarify exactly which tasks were blinded to intervention allocation (i.e., caregiving, outcome assessment and/or data analysis) and how.

***Item 11b. If relevant, describe the similarity of interventions.***

**Example:**

“*The placebo contained milk sugar and looked similar to the pimobendan capsules.”* (86)

“*The test treatment was identical to the commercially available product; the control treatment was similar in all aspects, except that it lacked the enteric coating, and was not commercially available. However, the organoleptic properties were identical and both treatments were presented in similar plain packaging (see below), ensuring that test and control treatment could not be distinguished.”* (37)

**Explanation:**

Blinding can be enhanced by ensuring that the interventions appear identical (76). For biologics and pharmaceuticals, this may involve creating similar looking substances (as in the examples provided) or vials with the same colored liquid. For surgical vs. medical interventions, this may involve the use of bandaging to conceal whether an incision was made. For nutritional interventions, this may involve pelleting diets to ensure an identical appearance. If it is not possible to make the appearance of the interventions similar, this should be explained and objective outcomes should ideally be used for determining response.

#### Statistical methods

***Item 12a. Describe the statistical methods used to compare groups for primary and secondary outcomes.***

**Examples:**

“*The primary outcome measure of clinical response rate regarding quality of life on M3 was evaluated according to a 4-level scale grid (clear improvement, improvement, insufficient improvement or failure). Both “clear improvement” and “improvement” levels were considered response to treatment. “Insufficient improvement” and “failure” classes were considered non-response (**Table 1**). A non-inferiority approach was used to compare the response rates, by calculating the odds ratio (OR) and its 95% onesided confidence interval. … For the secondary outcome measures, survival curves were compared using the Log-rank test.”* (87)

“*As a primary end point, the 95% CI of the CMPS-SF scale was used to compare dexketoprofen to methadone with a noninferiority test. Accordingly, a delta of 20% was considered a priori. In addition, according to clinical criteria and other published data, the delta values of secondary end points were 1.5 cm for DIVAS (reference), 3 N for MWTm and MWTw (20% of 15 N) and 2.2 ng dL*−*1 for cortisol (reference).”* (88)

**Explanation:**

A complete and accurate description of statistical analyses allows the reader to assess the validity of the statistical methods and the probability that analytical bias affected the internal validity of the study. The statistical analysis of RCT data should follow logically from the design of the study and should be pre-specified in the study protocol. Any additional analyses conducted that were not pre-specified in the protocol should be described as *post-priori*. The description of the analysis should start with identification of the population used in the analysis.

The authors should specify whether the analysis was based on intention-to-treat or per-protocol analysis. The intention-to-treat analysis, where intervention groups are compared as planned a priori regardless of deviations from the intervention as described in the protocol, maintains the statistical power of the trial, ensures the groups are comparable and that the results resemble clinical practice by assessing the effectiveness of the treatment. Per-protocol analysis is a non-randomized observational comparison of the animals that completed the protocol as planned. This may not represent a real-life situation because the difference observed between interventions may not be a function of the intended intervention only (i.e., it may be confounded by other factors). Often this analysis gives an exaggerated assessment of treatment effect. Per-protocol analysis requires adjustment for pre-randomization and post-randomization prognostic factors, and therefore the methods of analysis may differ substantially from the intention-to-treat analysis (89, 90). Intention-to-treat analysis is the preferred approach for superiority trials, with both analyses recommended for equivalence and non-inferiority trials (32, 71).

The approach to missing outcome data should also be described, because many options exist to account for missing data (91–94). The researchers should describe the analysis, including design features such as the unit of allocation, the unit of outcome measurement and the data form of the outcome measure (e.g., binary, ordinal, continuous, count). The purpose of the study (assessing superiority, equivalence or non-inferiority) and potential sources of non-independence between observations (including repeated measures over time or group housing of animals) should also be described.

Guidelines are available for reporting of statistical methods (95). Consultation with a statistician in the design and analysis stage of a clinical trial is strongly recommended. Authors should provide details of all descriptive and statistical modeling, which should include the name and data form of the outcome, the name and data form of the intervention, and other variables included in the analysis. Authors should name the test(s) used, for example, as *t*-test, chi-square test for proportions, Fisher's exact test, Mann-Whitney test or others, as well as the software used for the analyses. If the method is novel, a reference for the approach should be provided. If logistic regression modeling, the level of the outcome being modeled should be described (e.g., “*We modeled the probability of a positive outcome event”*). For categorical intervention variables, the referent should be clearly stated (e.g., “The *referent level of the intervention was Intervention A”*).

***Item 12b. Describe the methods used for ancillary analyses, such as subgroup analyses and adjusted analyses; report if these were pre-specified in the protocol or unplanned.***

**Examples:**

“*A repeated measures anova was initially considered for evaluation of differences between fluid groups (for all cats) for blood sodium, potassium, chloride, venous pH, and bicarbonate, but due to variation of the number of measurements in each time period, as well as heteroscedasticity, this could not be used. Therefore, we could only evaluate differences between groups using pairwise comparisons using the unpaired T-test or the Wilcoxon rank sum test depending on whether the data were parametric or nonparametric, respectively.”* (96)

“*As the severity of the disease at the time of enrolment can negatively affect the efficacy outcomes [reference], further statistical analyses were carried out on a subset of the PP population including only dogs with initial CSS* ≥ *8 to assess the effect of treatment in dogs with more severe clinical signs of OA.”* (97)

**Explanation:**

The approach to analyses should be pre-specified in the study protocol. However, additional analytic approaches may be conducted for several reasons; these include addition of a per-protocol analysis *post-hoc* (due to unanticipated non-compliance), adjusted analyses, and evaluation of subgroups (based on the results obtained in the preplanned analysis). Regardless of the reason, such analyses should be clearly labeled as unplanned or exploratory analyses, because the probability of spurious results increases with multiple analyses. For each additional analysis, the rationale should be provided along with the same level of detail required for the primary analysis. When adjusted analyses are conducted, such as a per-protocol analysis or because of imbalances in known confounders despite randomization, these analytical approaches are essentially treating the trial as a non-randomized observational study, and the inferences should be discussed as such.

### Results

#### Study subject flow

***Item 13a. For each group, state the number of study units (body part, individual animal, or litter) that were assessed for eligibility, randomly assigned, received the intended intervention, and were analyzed for each primary and secondary outcome.***

**Examples:**

Figure 1. (98)

**Figure 1:**
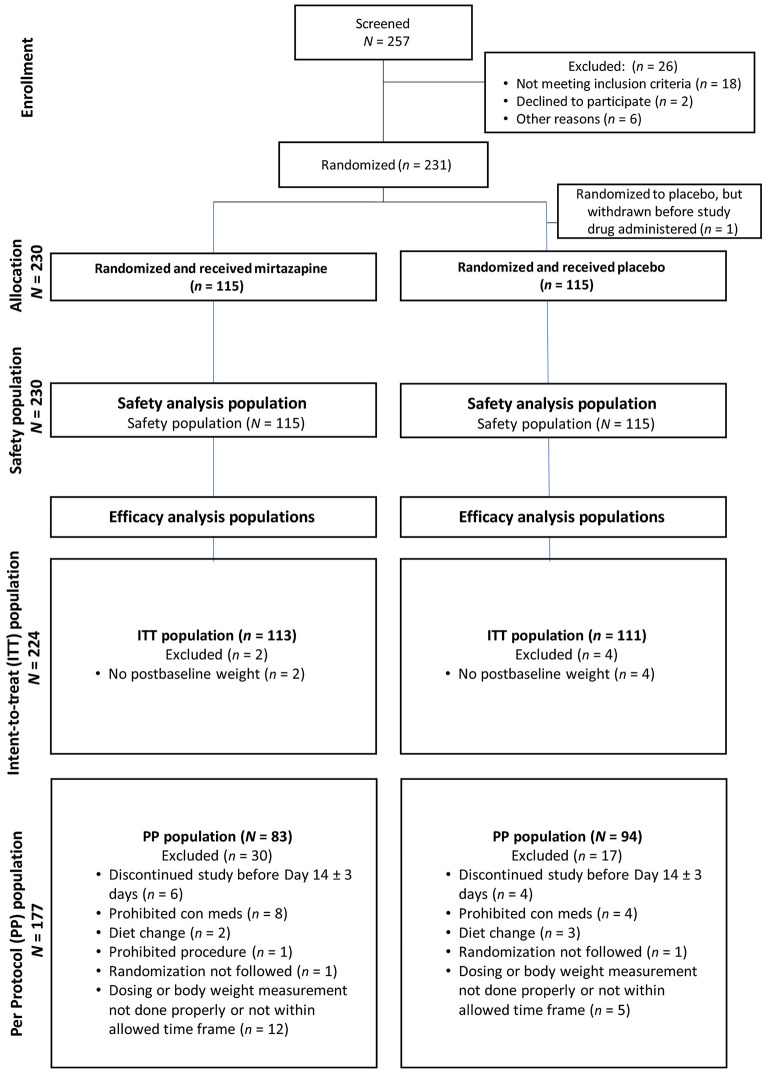
In this study, there were 257 cats screened; 26 were excluded and ultimately 231 were randomized to treatment. The Safety Population consisted of all cats randomized to either treatment group who received at least one dose of study drug. The Intent-to-Treat (ITT) population included all cats randomized and who received at least one dose of study drug and had at least one postbaseline body weight measurement. The Per Protocol (PP) population included all cats completing the study through and including Day 14. One cat with pre-existing dental disease underwent dental prophylaxis, and this cat was not included in the effectiveness population.

**Explanation:**

Clear understanding of the unit of study and the total number of study units that were eligible, enrolled, received the intervention as planned, were lost to follow up, or were excluded from analyses is crucial for internal and external validity of the trial to be evaluated. This information can be effectively described in the text for trials of short duration with no protocol failures or losses. However, for complex trials, or ones in which protocol deviations or losses have occurred, authors should strongly consider including a flow chart to describe study unit flow through the trial. Templates for flow charts are freely available from the CONSORT group (http://www.consort-statement.org/consort-statement/flow-diagram). Losses to follow-up and protocol failures (i.e., non-compliance with intervention protocol), which affect internal validity, should be distinguished. Knowledge of the total number of subjects included in analyses as compared to the total number originally allocated is necessary in order to assess the impact of those losses on the estimated effectiveness of the intervention. Further, the total number of subjects assessed for trial eligibility should be reported as it is relevant to the external validity of the trial (99).

***Item 13b. Quantify and explain any losses and exclusions after randomization for each group (such as the number per group removed due to adverse events) and for each intervention period in a crossover trial.***

**Examples:**

“*Five dogs were withdrawn prematurely from the study. Two dogs receiving the supplement were withdrawn due to infection prior to Day 42, one dog receiving placebo was withdrawn due to a fractured molar prior to Day 84, and one dog receiving the supplement was rescued for progressive pain associated with intervertebral disc disease at Day 68. One dog receiving the supplement had acute liver enzyme elevation at Day 42, therefore was withdrawn due to the potential of an adverse event*.” (100)

“*Data from 16 of the 17 dogs (9 in the CBD group and 7 in the placebo group) that completed the study were included in the analysis portion of the study. The owner of the remaining dog (in the placebo group) reported giving the dog CBD-infused oil during the final month of the study; therefore, that dog was excluded from analysis*.” (101)

**Explanation:**

All study subjects excluded after randomization should be reported. If a flowchart was used to describe study subject flow through the trial (Item 13a), it may be possible to include the nature of the protocol deviation within the diagram. The flowchart can also be used to enumerate study subjects that were lost due to adverse events after randomization. However, the exact reason for exclusion should be reported and the use of vague terms like “protocol deviation” does not provide sufficient justification for post-randomization exclusion to readers.

#### Recruitment

***Item 14a. Report the dates defining the periods of recruitment and follow-up.***

**Examples:**

“*The first dog was enrolled on 2 October 2015, and the last dog completed the in-life phase of the study on 11 October 2016*.” (102)

“*All patients that underwent a TPLO as treatment for cranial cruciate ligament disease with or without concurrent patellar luxation between July 2015 and September 2016 were enrolled… Active surveillance, defined as direct examination by either the surgeon (Diplomate, resident or surgery intern) or referring veterinarian, was performed at 30 and 90 days postoperatively*.” (103)

**Explanation:**

Due to the continuous evolution of medical and surgical therapies, alerting readers to the dates when subjects were recruited and when the trial occurred helps place the study into the correct historical context (34). If follow-up of study subjects ended on a specific date, leading to different time at risk for study subjects, the date of trial cessation should be reported along with the minimum, maximum, and median duration of follow-up (104). Any differences in the dates when recruitment occurred for the control and intervention groups should be explicitly noted. Likewise, any differences in the start or end dates for any groups included in the study should be reported.

***Item 14b. If the trial was discontinued early, provide the reason.***

**Examples:**

“*The high number of excluded cats was unexpected, and the study was terminated when 30 cats per group were reached due to time and budget constraints*.” (41)

“*Because of the important number of side effects observed, the study was terminated at 40 instead of 80 dogs*.” (105)

**Explanation:**

There are several scenarios that could lead to a trial being discontinued early. Broadly, trials may be discontinued due to safety concerns for the study subjects or their caregivers, obvious benefits of one study arm as compared to another, or if the hypothesis is shown to be untestable within the constraints of the trial based upon results of an interim analysis (106). As mentioned above (item 7b), there is evidence that trials stopped early may report greater effect sizes than trials on the same subject that are not stopped early, representing a potentially serious bias. Trials stopped early also result in less opportunity for detecting adverse effects. Therefore, if a trial is stopped early, the specific reason for discontinuation of the trial should be reported (75). Any factors relevant to the decision to stop the trial should be noted.

#### Baseline data

***Item 15. Provide a detailed description (such as a table) of baseline demographic and clinical characteristics that could impact the outcomes for each intervention group.***

**Examples:**

Table 2. (107)

**Table 2:** Clinical and clinicopathological parameters in hyperthyroid cats treated for a 3 months period with iodine-restricted food, transdermal methimazole, or oral methimazole [adapted from Grossi et al. (107)].

**Variable**	**Group A: Iodine-restricted food**	**Group B: Transdermal methimazole**	**Group C: Oral methimazole**
**Baseline data (T0)**			
No. of cats	13	11	9
Body weight (kg)	3.3 (2.0–5.3)	3.8 (1.9–4.3)	3.5 (2.5–5.4)
TT4 (nmol/l) (RI:15–42)	156 (98–309)	225 (89–309)	142 (58–309)
Creatinine (mol/l) (RI:70–159)	93 (55–163)	96 (69–150)	102 (84–143)
Urea (mmol/l) (RI:10.7–23.2)	20.1 (15.9–37.9)	23.6 (12.9–37.0)	21.4 (12.9–31.2)
ALT (U/l) (RI:22–45)	164 (27–708)	185 (48–1,130)	110 (58–591)
AST (U/l) (RI:14–41)	54 (23–228)	65 (25–141)	49 (26–186)
ALP (U/l) (RI:0–120)	285 (41–537)	182.5 (25–420)	83 (45–447)

**Explanation:**

It is important to summarize the characteristics of the study population and groups for internal and external validity to be evaluated. Reporting baseline data allows clinicians to determine the relevance of the study results for their practice or for an individual patient. Providing this information also allows readers to assess the comparability of groups by comparing demographic and clinical characteristics relevant to the population or intervention. In addition, randomization should minimize the chances that the groups will differ with respect to important prognostic factors, but it does not ensure that intervention groups will be equivalent at baseline. Therefore, it is important to provide information on baseline characteristics by group to allow readers to evaluate whether the populations were comparable, and whether the distribution of prognostic variables is equal between groups, or whether residual confounding is likely to be present.

Baseline data are often easier for the reader to assess if they are presented in a table. It is not appropriate to test for statistically significant differences between groups at baseline, because differences found after randomization are the result of chance rather than bias (108). However, imbalances may occur even after randomization, particularly for small sample sizes. Rather than comparing groups based on statistical testing, differences between groups at baseline should include a consideration of the prognostic strength of the factor and the magnitude of any chance imbalances (109). Average values and variability within the data for continuous variables, such as weight, should be reported. Typically, this may include the mean and standard deviation for each group, but medians and percentile ranges may be preferable if the continuous data have an asymmetrical distribution (34). Ordinal categories, such as stages of disease, and nominal categories, such as breed and sex, should be reported using numbers and proportions for each category (110). Standard errors and confidence intervals should not be used when measuring or describing variability because they are inferential statistics. For nutrition-related studies, body weight, body condition score, and muscle condition score should be reported.

#### Numbers analyzed

***Item 16. Report the number analyzed for the primary and all secondary outcomes and whether the analysis was by original assigned groups (intention-to-treat) or per-protocol. Explicitly report the numbers of units lost to follow-up and, if relevant, the number of animals with changed intervention assignments (if relevant for per-protocol).***

**Example:**

“*Sixty dogs met entry criteria. Seven were disqualified because of study protocol violations including missed immediate postoperative period treatment (n* = *3), damage to the treatment device from patient chewing (n* = *2), medical complication of myelomalacia (n* = *1), and failure to obtain incisional photographs on day 0 (n* = *1). The final study population included 53 dogs; 27 were in the PEMF (test) group and 28 were in the control group*.” (20)

“*The Intention To Treat (ITT) population included all animals that were randomized and received at least one dose of study treatments. The Per Protocol (PP) population included dogs that were fully compliant with the protocol except for cases with minor deviations that would not affect the results”* (97)

**Explanation:**

In the Results section, the authors should explicitly state for each outcome the exact number of participants included in each group. It is important to report the number per group for each outcome because the numbers included in the analysis may differ among different outcomes. The flow chart (Item 13) might only include the primary outcome and a subset of the secondary outcomes. It also is possible that when both an intention-to-treat and a per-protocol analysis are conducted, the numbers might differ, because non-compliant individuals may be included in the intention-to-treat analysis, but excluded from the per-protocol analysis, or included in the intervention group corresponding to the intervention received rather than the allocated intervention.

#### Outcomes and estimation

***Item 17a. For each primary and secondary outcome, report the results for each group, and the estimated effect size and its precision (such as 95% confidence interval).***

**Examples:**

“*Dogs receiving prednisone and prednisone/aspirin had 11.1 times (95% CI, 1.7-73.6) and 31.5 times (95% CI, 3.5-288.0) higher odds, respectively, of having endoscopic mucosal lesion scores* ≥*4 than dogs receiving placebo (P* ≤ *0.01).”* (111)

“*The pheromone* + *insecticide intervention provided 13% (95% C.I. 0%, 44.0%) protection against anti-Leishmania antibody seroconversion, 52% (95% C.I. 6.2%, 74.9%) against parasite infection, reduced tissue parasite loads by 53% (95% C.I. 5.4%, 76.7%), and reduced household female sand fly abundance by 49% (95% C.I. 8.2%, 71.3%)*.” (112)

“*At the primary efficacy assessment on day 28 or 42, 92.4.0% (109/118) of the cefovecin group and 92.3% (108/117) of the cefadroxil group were considered treatment successes (referenced table). The noninferiority test conducted to compare percentage of dogs in the 2 treatment groups that were considered a clinical success revealed that cefovecin was overall (ie, independent of clinical diagnosis) noninferior to cefadroxil in the treatment of these skin infections. Additional analyses revealed cefovecin treatment to be noninferior to cefadroxil treatment for each of the 3 clinical diagnoses (ie, bacterial folliculitis, wound, and abscess).”* (113)

**Explanation:**

The results of all analyses for each primary and secondary outcome in a trial should be reported, including the results for each intervention group, the estimated magnitude of differences between groups (effect size), and the precision (e.g., 95% confidence interval). This information is essential for the reader to interpret the clinical significance in addition to any statistical significance of the reported difference between the intervention groups. This is a more informative approach than reporting of *p*-values which does not convey any information about precision (114) and therefore reporting of *p*-values alone is insufficient. For continuous variables, the results of each group mean, standard deviation, and the effect size (the mean difference and 95% CI) should be reported. For categorical variables, results should be expressed as the number that had the event of interest [with the number at risk of this event (i.e., the denominator in proportion or percentage calculations) also reported if not already clear], proportion or percentage, and the effect size (e.g., risk ratio, odds ratio, risk difference) and its measure of precision (e.g., 95% CI).

***Item 17b. For binary outcomes, present both absolute and relative effect sizes.***

**Examples:**

“*Dogs in the ADPP group had a significantly shorter duration of diarrhea (ADPP: median, 32 hours; 95% confidence interval [CI], 2-118; n* = *51; Placebo: median, 47 hours; 95% CI, 4-167; n* = *58; P* =*.008) and the rate of resolution of diarrhea was 1.60 times faster in the ADPP group than in the Placebo group (ratio, 1.60; 95% CI, 1.08-2.44; P* = *0.02)*.” (115)

“*Cumulative number (%) of cats with recurrent UO at 10 days, 1-, 2-, and 6-months after discharge was 1 (2%), 2 (4%), 4 (8%), and 8 (16%), respectively…. No difference in the cumulative incidence of UO within 6 months was detected with addition of meloxicam (odds ratio [95% confidence interval], 0.63 [0.13-2.97]; P* = *0.70)*.” (116)

**Explanation:**

To provide comprehensive information about intervention effects, both absolute and relative effect sizes should be reported. Relative effects compare one intervention group with another for a single measure, such as risk ratio, hazard ratio, or odds ratio; absolute risk measures describe the probability that an outcome will occur, and include measures such as absolute risk and risk difference (72, 117). The presentation of relative effect sizes (e.g., risk ratios) enables readers to compare the occurrence of an event of interest between groups. An advantage of using relative measures is that they are expected to be stable across different study populations with different baseline risks of the outcome (118). However, relative effects do not provide an indication of baseline risk and, therefore, might be less relevant to clinicians and animal caregivers. Further, relative measures can lead to overestimation of the effect of an intervention. The absolute effect size (e.g., risk differences) highlights the difference in risk between groups, giving the reader a better representation of the actual situation (117). For example, if the relative risk of mortality following an intervention was 2, this would differ in importance if the mortality rates were 2 vs. 1% or 40 vs. 20%.

#### Ancillary analyses

***Item 18. Present the results of any other analyses performed, including subgroup analyses and adjusted analyses, distinguishing pre-specified from unplanned or exploratory analyses.***

**Examples:**

“*Given the small number of patients admitted with SE (status epilepticus) in both groups, statistical analysis was performed taking into consideration only patients affected by CS (cluster seizures)*.” (119)

“*The possible confounding effects of the intraoperative administration of hydromorphone on recovery were evaluated. Twelve dogs in group P and 10 dogs in group KD did not receive hydromorphone at any time during the anesthetic period. The time elapsed from the last dose of intraoperative hydromorphone to the end of anesthesia differed significantly between the groups (P* = *0.02) with group P and group KD receiving the last dose (mean* ± *SD) 55.88* ± *13.4 and 115* ± *18.5 minutes before the end of anesthesia, respectively*.” (120)

**Explanation:**

The results from all analyses need to be reported, including reporting pre-specified analyses, unplanned or exploratory analyses, any subgroup analyses and adjusted analyses. It is important to make readers aware of the number of analyses performed because an increased number of analyses of the same data increases the risk of type 1 errors (false-positive findings) (121). This is of particular concern for clinical trials with multiple outcomes. Conversely, additional outcomes or subgroup analyses might be at risk of type II error because the power of the study is determined with reference to the primary outcome measure. Results of all subgroup analyses that were performed should be reported, otherwise bias could result from selective reporting, and unplanned subgroup analyses should be distinguished from subgroup analyses that were pre-specified in the protocol. Given that spurious results are more likely with unplanned analyses, including subgroup analyses (122), reporting of unplanned analyses enables readers to interpret trial results appropriately (123). Results from tests of interaction (item 12b) should be reported as estimated differences in the intervention effect of each subgroup, along with the relevant confidence interval. Authors should state whether tests of interactions were pre-specified. If adjusted analyses are performed, the unadjusted results should also be provided. If preplanned analyses were not performed, the reasons for this should be explained.

#### Harms

***Item 19. Describe the methods for detection of adverse events and report all adverse events (expected, unexpected, and suspected) or unintended effects observed in each group or their absence.***

**Examples:**

“*[Methods] Every dog had its catheter site evaluated for evidence of phlebitis every 6 h by shifting the neck bandage down and exposing the CVC insertion site. Phlebitis was defined as the presence of any of the following: erythema, tenderness, swelling, unusual discharge, or warmth …. [Results] Subjective signs of mild phlebitis were observed in two dogs in the S group at 54 and 72 h, respectively, and one from the HS group at 48 h.”* (124)

“*During the treatment period, vomiting was recorded on 1 day for 3 dogs (1, aspirin; 2, prednisone/aspirin), on 2 days for 1 dog (aspirin), and on 3 days for 1 dog (placebo). Neither hematemesis or melena nor hematochezia occurred during the study*.” (111)

**Explanation:**

It is common during the course of a trial for unintended effects to occur. Knowledge of both these potential harms and the benefits of interventions is necessary to enable readers to make rational, evidence-based decisions. For companion animals, adverse events include those that affect animal health, welfare, appearance, behavior, or performance.

Although detection of rare or long-term harms is unlikely during a RCT, this type of study can provide some information about common or short-term adverse effects. Some adverse events that occur during a trial may be a consequence of the condition being treated rather than the intervention being tested. It is important that authors provide estimates of the frequency of adverse events noted during the trial regardless of the underlying cause. In cases where the intervention was discontinued or where there were reductions in dosages, reasons should be explicitly stated for each occurrence.

In response to incomplete reporting of harms in human trials, a CONSORT extension for reporting of harms is available (125). Data related to adverse events should be reported separately for each intervention group in the trial. The methods used for data collection for adverse events should also be explicitly described, including detection methods, adverse event definition (and whether it is based on a validated measure), and grading when applicable. State whether recurrent events (the occurrence of the same adverse event more than once in the same participant) are counted as separate events or as a single event should also be stated. At a minimum, estimates of the frequency of adverse events and reasons for intervention discontinuation and dosage reductions should be provided for each intervention group. A balanced discussion of benefits and harms should be provided by the authors (125). If no adverse events were detected, a statement should be included to this effect.

### Discussion

#### Interpretation

***Item 20. Ensure that interpretation is consistent with results, balancing benefits and harms, and considering other relevant evidence.***

**Examples:**

“*Hyperthyroid cats medicated with 20 mg/kg gabapentin 1 h prior to leaving home were more relaxed during transport and more compliant with veterinary procedures than cats administered a placebo solution. These findings are in agreement with work in healthy cats [reference]… Despite the flavoring, the bitterness of gabapentin is difficult to disguise. The solution was well tolerated by the majority of cats. One cat vomited after administration of the solution and was removed from the study.”* (126)

**Explanation:**

The key result(s) of the study should be highlighted in the Discussion section. These key results should be put in the context of the reported literature and compared with the results of previous trials. Ideally, key results are best undertaken by comparing to a recent systematic review if available (127). Systematic reviews allow assessment of the trial results and comparison of participants across trials. When a systematic review has not been undertaken, the discussion should be based on a focused and transparent literature search, rather than selectively including studies (for example, only those that supported the current trial results). Although a trial may present evidence that an intervention is beneficial, a consideration of potential or observed harms also should be considered when presenting an interpretation of trial results.

#### Generalizability

***Item 21. Discuss generalizability (external validity, applicability) of the trial findings.***

**Example:**

“*To our knowledge, this study is the first prospective block randomized study to assess plaque accumulation performed within a general practice setting and the first to compare three commonly used plaque control methods concurrently. These elements improve the internal validity, external validity/generalisability and relevance of the results to the general practice dog population.”* (128)

**Explanation:**

The external validity or applicability of a trial refers to the extent that the trial results can be generalized to other situations (129). Generalizability will be enhanced by minimizing the potential for selection bias resulting from a mismatch between animals in the trial and animals in the target population. The extent to which the design and conduct control bias is considered internal validity, and such validity is critical to avoid flawed trial results that render any external validity or generalizability irrelevant (130). Assessment of the generalizability or external validity of a trial is a clinical rather than a statistical judgement (130). External validity can be affected by various factors, including the animals' characteristics, the trial setting, the intervention regimens tested, and the outcomes assessed (130). For example, can the results be generalized to an animal that differs from those that participated in the trial by age, sex/neuter status, breed, disease severity or co-morbidities? Are the results applicable to animals in general practice, vs. referral practice, or in different countries or continents? Not only should authors discuss their interpretation of the external trial validity, they should also provide adequate information for readers to assess external validity themselves. The information required to assess external validity should have been comprehensively reported for the items related to eligibility criteria (Item 4a), trial setting and location (item 4b), interventions and administration methods (item 5) outcome definitions (item 6a), and the recruitment and follow-up periods (item 14a).

#### Limitations

***Item 22. Discuss trial limitations, addressing sources of potential bias, imprecision, and, if relevant, multiplicity of analyses. Consider potential carryover effects if a crossover trial.***

**Example:**

“*A limitation of the present study was the exclusion of cats with SABP above 200 mmHg (ie, cats with most severe systemic HT and highest risk of TOD) as it was considered unethical to enroll such cats where the risk of TOD appears to be extremely high into a placebo-controlled study. In accordance, the primary end point criterion that the mean SABP reduction should be* ≥*20 mmHg would not necessarily lead to a reduction in the category of risk for future TOD if cats with SABP above 200 mmHg would have been included.”* (131)

“*One relevant limitation of this clinical study could be the study center effect. However, as the number in each of the centers was rather small and each dog experienced at each visit the same scenario, a center effect was expected to be small and was not identified on statistical analysis. We were also not able to identify a treatment order bias. In addition, the findings of the current trial confirm earlier findings in a single center study. Another limitation of the analysis was that an intention to treat analysis (ITTA) was not conducted. Normally, ITTA is an analysis approach in which all recruited patients are evaluated as block randomized, regardless of the dietary intervention they actually received. However, as in randomized clinical trial with epileptic patients, the dropout rate appears to be higher, especially in the beginning, all of the 8 dogs dropped out in the first leg of the study. Therefore, an ITTA would not be appropriate.”* (132)

**Explanation:**

Identification and discussion of weaknesses of a trial is an important component of a discussion section (133). Some limitations may be related to the design of the study, such as if a validated scale for measuring the outcome was not available, or if the investigators used biomarkers rather than clinical outcomes to reduce the necessary sample size because of funding limitations. Some limitations might be unforeseen issues that occurred during the conduct of the study, for instance if problems with recruitment arose. The discussion of study limitations should include a consideration of the potential for bias (including selection bias, information bias, and confounding). The authors also should address precision, including any implications for study power if the *a priori* assumptions for calculating the sample size differed from the actual values in the trial (e.g., differences in baseline prevalence or variability in a continuous outcome from those used in the sample size calculation) (133). Authors should consider the difference between clinical significance and statistical significance, and should consider the total set of effect sizes with which their data are plausible and avoid interpreting non-significant results as evidence of equivalence of interventions, unless the trial was designed to demonstrate non-superiority or equivalence (34). Authors also should consider the increased potential for type I errors when multiple statistical comparisons of the same outcome were evaluated.

The limitations section should include a thoughtful consideration of the anticipated effect that any issues might have had on the study results, rather than an extensive list of possible limitations or an unsubstantiated statement that the limitations did not matter. The Limitations section should not be used to justify poor study design, but rather to provide the reader with information that will help them to interpret the results of the trial.

### Other information

#### Registration

***Item 23. State whether the trial was registered and, if so, provide a registration number and name of trial registry. If not, provide a reason for not registering the trial in advance.***

**Example:**

We were not able to find an example in the veterinary literature. Therefore, we present here the example from the CONSORT 2010 Elaboration document (34).

“*The trial is registered at* *ClinicalTrials.gov**, number NCT00244842.”* (113)

**Explanation:**

Trial registries are a forum whereby researchers can publicly report information on the design, and administration of a clinical trial, prior to commencement of the actual trial. Having a record of trials that are recruiting or completed provides transparency in planned outcomes, allowing readers of trials to evaluate selective outcome reporting, and also provides a means of evaluating publication bias. The World Health Organization, which runs the International Clinical Trials Registry Platform, states that “the registration of all interventional trials is a scientific, ethical and moral responsibility” (134). The International Journal of Medical Journal Editors stated that trials started after July 2005 should be registered prior to patient enrollment to be considered for publication in member journals (135). However, in an evaluation of trials in dogs and cats, none of the trials included information on trial registry (18). The American Veterinary Medical Association (AVMA) maintains a trial registry (the AVMA Animal Health Studies Database) (136) that serves both to aid in patient recruitment and as an archive of proposed trial methods (137). Authors who have registered their trial in this (or another) registry should state the name of the registry and the registration number assigned to the trial. Trial registration is encouraged and therefore, if a trial is not registered, the authors should explicitly state this and provide a justification for not registering the trial.

#### Protocol

***Item 24. State if the full trial protocol was finalized a priori and where it can be accessed. Describe any protocol deviations with justification.***

**Example:**

“*The experimental protocol is outlined in Supplemental Table 1*.” (138)

**Explanation:**

Creating a pre-planned protocol for the proposed methods for a trial (as opposed to a protocol for a specific procedure within a trial) is important because it provides an *a priori* record of the methods of the trial, including specification of the primary and secondary outcomes. This helps to prevent selective outcome reporting based on the statistical significance of results. There are a number of ways that a protocol can be made accessible. These include inclusion as supplementary material to the full trial publication, posting of protocols on researchers' webpages or institutional repositories, publication in a peer-reviewed journal, or having a time-stamped copy available on request of the authors. Any changes to the protocol after initiation of the trial should be stated in the full report from that trial, with a justification. This includes changes to eligibility criteria, interventions, outcomes or their measurement (item 6b), sample size, data management, or analytical methods.

#### Funding and transparency

***Item 25. State sources of funding and other support (such as supply of drugs), role of funders, conflict of interest, ethical approval for human (if applicable) and animal subject use, and quality standards used.***

**Examples:**

“*Regivet supplied the midazolam used in this study. They played no role in the study design or in the collection, analysis, and interpretation of data…The study protocol was approved by the University Animal Ethical Review Committee (VIN/15/033), and informed owner consent was obtained for all dogs enrolled in the study…The study was also conducted under an Animal Test Certificate (42273/003) and complied with Good Clinical Practice standards.”* (105)

“*Dr. Langlois serves on a scientific advisory board for Zomedica, Inc. The authors declare no additional conflicts of interest.”* (139)

**Explanation:**

Studies have shown that trials comparing pharmaceuticals or biologics are more likely to produce results that favor products made by the company sponsoring the research than when trials are funded through other sources (140–142). Veterinary trials with pharmaceutical company funding or involvement were more likely to report positive findings compared to trials funded through other sources (143). Thus, all sources of funding or support (including supplying drugs or contributing to the writing of the manuscript) for a trial should be reported in order for readers to assess the validity of the findings. If the funder had an active role in the design of the study, analysis of the data, or decisions to publish the results it should be explicitly stated. Likewise, if the funder had no such involvement, it should be explicitly reported.

## Data availability statement

The original contributions presented in the study are included in the article/supplementary material, further inquiries can be directed to the corresponding author.

## Consensus group members

Members of the Consensus Group are (in alphabetical order) Karin Allenspach (Iowa State University), David J. Argyle (The University of Edinburgh), Cynthia Bashore (The US Food and Drug Administration), Erika Berger (National Institutes of Health), Philip J. Bergman (VCA, Clinical Studies), Adrian Boswood (The Royal Veterinary College), Benjamin M. Brainard (University of Georgia), Marnie L. Brennan (University of Nottingham), Dave Brodbelt (The Royal Veterinary College), Jeffrey N. Bryan (University of Missouri), Steven Budsberg (University of Georgia), Jenna H. Burton (Colorado State University), Daniel L. Chan (The Royal Veterinary College), Michael G. Conzemius (University of Minnesota), William S. Dernell (Washington State University), Nicola Di Girolamo (Cornell University), Richard Evans (Thomas Jefferson University), Aiden P. Foster (University of Bristol), Lisa M. Freeman (Tufts University), Alexander J. German (University of Liverpool), Michelle A. Giuffrida (University of California, Davis), Wanda J. Gordon-Evans (University of Minnesota), Nicolas Granger (Highcroft Veterinary Referrals), Laura L. Hungerford (Virginia Tech), Nick Jeffery (Texas A&M University), Unity Jeffery (Texas A&M University), Chad M. Johannes (Colorado State University), Aarti Kathrani (The Royal Veterinary College), Susan Lana (Colorado State University), Amy K. LeBlanc (National Institutes of Health), Sandra L. Lefebvre (American Veterinary Medical Association), Dana N. LeVine (Auburn University), Christopher Loss (The US Food and Drug Administration), Caroline Mansfield (University of Queensland), Philipp Mayhew (University of California, Davis), Sarah A. Moore (The Ohio State University), Ralf Mueller (Ludwig-Maximilians-University of Munich), Allison L. O'Kell (University of Florida), Dan O'Neill (The Royal Veterinary College), Natasha Olby (North Carolina State University), Thierry Olivry (North Carolina State University), Rodney L. Page (Colorado State University), Jessica M. Quimby (The Ohio State University), Robert B. Rebhun (University of California, Davis), Carolina H. Ricco Pereira (The Ohio State University), Courtney Shaw (The US Food and Drug Administration), Douglas H. Thamm (Colorado State University), David M. Vail (University of Wisconsin-Madison), Kristen M. Weishaar (Colorado State University), Constance N. White (Oregon State University), Alexandra L. Winter (Merck), and Luke A. Wittenberg (University of California, Davis).

## Author contributions

AR, JS, AO'C, and LS comprised the steering committee for this initiative. The steering committee led the development of the reporting item checklist and created an initial draft of this elaboration document. All members of the consensus group provided input and edits to this document. AR oversaw communications with the consensus group. AR and JS collated comments of all authors into the final manuscript draft. All authors contributed to the article and approved the submitted version.
